# Efficacy of Locally Delivered Aloe Vera Hydrogel in Patients With Chronic Periodontitis: A Prospective Clinical Study

**DOI:** 10.7759/cureus.59109

**Published:** 2024-04-26

**Authors:** Chanchal Katariya, Arvina Rajasekar

**Affiliations:** 1 Periodontics, Saveetha Dental College and Hospitals, Saveetha Institute of Medical and Technical Sciences (SIMATS) Saveetha University, Chennai, IND

**Keywords:** aloe vera gel, periodontitis, scaling and root planing, local drug delivery, herb

## Abstract

Background: Various herbal and natural products have been used for multiple purposes in medicine due to recent interest and advancements in the field of alternative medicine. For the past few millennia, aloe vera has been used as medicine. Its anti-inflammatory and antibacterial properties have been proven to reduce periodontal disease.

Aim: In patients with generalised chronic periodontitis, this study examined the impact of aloe vera hydrogel in conjunction with scaling and root planing (SRP).

Methods: Sixty patients with generalised chronic periodontitis were enrolled in this study and split into two groups: Group 1 (control) - SRP alone (n=30) and Group 2 (test) - Aloe vera hydrogel with SRP (n=30). Clinical parameters related to periodontal disease, such as plaque index (PI), clinical attachment level (CAL), and probing depth (PD) were measured at baseline and three months after the procedure, and the results were compared using Statistical Product and Service Solutions (SPSS, version 23.0; IBM SPSS Statistics for Windows, Armonk, NY) software. A p-value of <0.05 indicated that the result was statistically significant.

Results: When comparing both groups' third-month periodontal clinical parameters to the baseline, there was a significant improvement (p<0.05). In the third month, the test group showed better improvement in PD and CAL than the control group (p<0.05).

Conclusion: The combination of SRP and aloe vera hydrogel greatly improved periodontal clinical parameters. However, studies with long-term follow-up assessing the efficacy of other modes of delivering aloe vera and also its effect on microbiological and immunological parameters are warranted in the future to substantiate these findings.

## Introduction

The resorption of alveolar bone and loss of soft tissue attachment are the signs of periodontitis, a disease that affects the tissues supporting the teeth. Dental plaque is thought to be the primary causative factor since it contains harmful microorganisms [1]. Treatment options for periodontal diseases include both surgical and non-surgical procedures. Mechanical debridement is non-surgical periodontal therapy, which is regarded as the gold standard procedure and is used to eradicate these disease-causing microbiota [2]. Nevertheless, mechanical treatment is ineffective in regions of furcation involvement and deep periodontal pockets. Because deep subgingival areas are difficult to access, periodontal pathogens cannot be completely eliminated, which raises the possibility of treatment failure [3]. For this reason, studies have shown that, with systemic antimicrobial therapy along with mechanical treatment, the disease progression is better and faster [4]. These antimicrobials have their own negative effects, such as superimposed infections, resistant strains, and poor patient compliance [5].

Local drug delivery (LDD) has become more significant recently in periodontology. It has been demonstrated that delivering the medication into the periodontal pocket reduces pocket depth, stabilizes the periodontal attachment, and reduces bleeding, allowing for better disease control. Due to its ability to reach the periodontal pocket's base and its capacity to be maintained for long enough for the antibacterial action to take place, LDD achieves bioavailability to surrounding tissues without causing any negative side effects [6]. Although a variety of microbial agents are frequently used as LDD in periodontology, the search for cost-efficient, safe agents has always led researchers to turn to herbal remedies [7]. Herbosomes are recently developed herbal formulations that absorb better than systemic antibiotic treatment, which produce greater bioavailability and activities. Due to its non-toxicity and compatibility with pharmaceutical formulation, herbal medicines have been utilized extensively throughout history.

One such herbal product is aloe vera, which belongs to the family of Asphodelaceae. Out of more than 400 species of aloe, Aloe barbadensis and Aloe arborescens are the two most commonly used for pharmacological, cosmetic, and medicinal purposes. Aloe vera has a complex composition, with 75 different substances, including minerals, enzymes, sugars, anthraquinones, lignins, saponins, sterols, amino acids, and salicylic acid found in its leaves, which contain 99.5% water and 0.0013% protein. Isorabaichromone, feruloylaloesin, and p-coumaroylaloesin are three aloesin derivatives that have demonstrated robust capacity to scavenge superoxide and free radicals [8]. In vitro and in vivo studies have demonstrated the anti-inflammatory, antibacterial, antioxidant, immune-boosting, and hypoglycemic properties of aloe vera gel [9]. Aloe vera has been used in dentistry for a number of purposes, including the treatment of periodontal disease [10], the sterilization of gutta-percha [11], intracanal medicament [12], the treatment of Candida albicans [13], and the treatment of aphthous stomatitis [14]. The current study was done to assess the effectiveness of aloe vera hydrogel in generalised chronic periodontitis patients as an adjuvant to scaling and root planing (SRP).

## Materials and methods

Study setting

This clinical research was carried out among 60 generalized chronic periodontitis patients in the Department of Periodontics and Implantology, Saveetha Dental College and Hospitals, Chennai, after getting approval from the Institutional Ethical Board (Ref- IHEC/SDC/PERIO-2001/22/058). Every patient provided signed informed consent. The sample size was determined to be 60 by taking the mean and standard deviation values from the previous study [15] using G*Power software (version 3.1.9.4; The G*Power Team, Germany). The participants were split into two groups: Group 1 (control) - SRP alone (n=30) and Group 2 (test) - aloe vera hydrogel with SRP (n=30). Patients aged between 25 and 65 years, systemically healthy individuals, and with generalized chronic periodontitis were included in the study. Smokers and tobacco users, pregnant ladies and lactating women, systemically compromised patients, patients who had undergone periodontal therapy before three months, and patients on any antibiotic therapy were excluded from the study.

Preparation of aloe vera hydrogel

**Figure 1:**
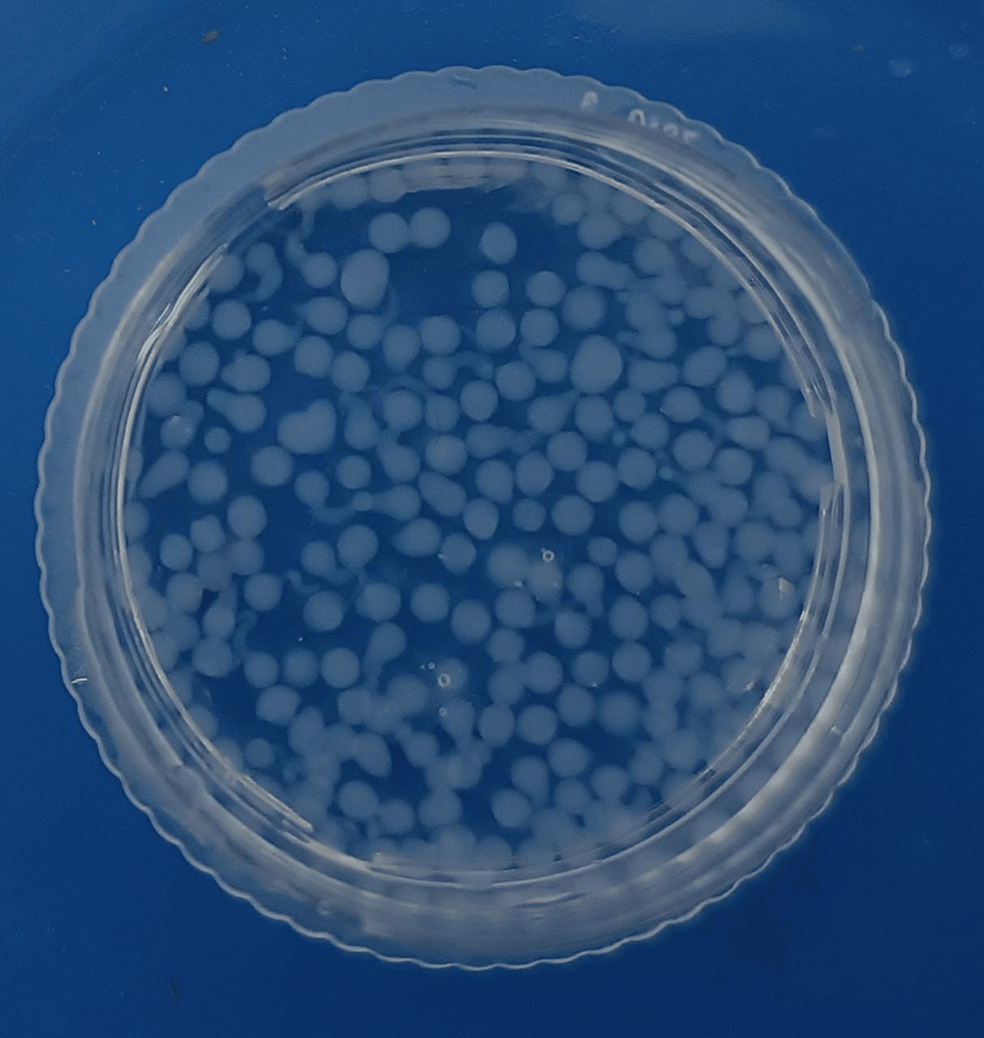
Aloe vera hydrogel

Aloe vera was obtained from ripe plant leaves, washed, and extracted after discarding the outer skin. The plant extract was homogenized using a blender and stored in a refrigerator. A 3% (w/v) concentration of sodium alginate was dissolved in ultrapure water (MilliporeSigma, Burlington, VT). Additionally, 1% (w/v) of aloe vera extract was added to the previously mentioned sodium alginate solution, and everything was well blended. Using an insulin syringe and continuous stirring at room temperature, the polymer solution containing aloe vera extract was then added dropwise into the gelation median of 200 mL of 6% (w/v) calcium chloride solution. The resulting microbeads were then removed from the gelation medium after 15 minutes and placed in a temperature-controlled chamber to dry (Figure 1). Throughout the experiment, the following parameters were maintained: the temperature, the number of drops that fell into the gelation medium per minute, and the amount of space between the syringe and the gelation medium.

Clinical procedure

**Figure 2:**
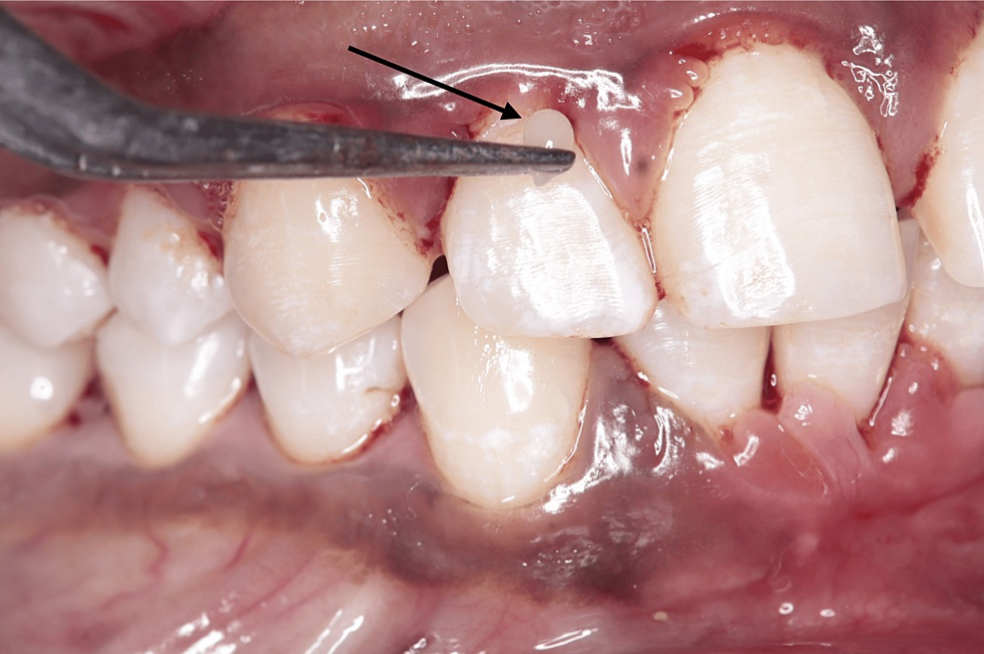
Clinical application of aloe vera hydrogel microbeads into the periodontal pocket

Both the group patients underwent SRP using sterile curettes (Hu-Friedy®, Chicago, IL). After receiving SRP treatment, the test group participants had a single application of aloe vera hydrogel microbeads into the periodontal pockets (Figure 2).

Outcome parameters

Probing depth (PD) from the gingival margin to the base of the sulcus, clinical attachment level (CAL) from the cementoenamel junction to the base of the sulcus, and Turesky modification of the Quigley Hein plaque index (PI) were recorded using a UNC-15 periodontal probe. At baseline and the third month, all the above-mentioned parameters were recorded.

Statistical analysis

Using Statistical Product and Service Solutions (SPSS, version 23.0; IBM SPSS Statistics for Windows, Armonk, NY), the data were analyzed. A paired t-test was used to compare all the parameters between various time intervals within each group, whereas an independent t-test was employed for intergroup comparison. A p-value of <0.05 indicated that the result was statistically significant.

## Results

In this clinical experiment, 30 patients were subjected to SRP alone (Group 1) and 30 patients to SRP with aloe vera hydrogel (Group 2). Following the intervention, none of the study participants experienced any clinically evident negative effects.

There was no significant difference between the groups at baseline in terms of PD (p = 0.78), CAL (p = 0.31), and PI (p = 0.67). After three months in both groups, there was a significant improvement in PD (p = 0.0), CAL (p = 0.00), and PI (p = 0.00). The intergroup comparison showed statistically significant improvement in the SRP + aloe vera hydrogel group as compared to SRP alone in terms of PD (p = 0.03) and CAL (p = 0.001), except for PI (p = 0.18) (Table 1).

**Table 1:** Between group and within group comparison *Significance: p < 0.05; #p value: Independent t-test; ## p value: Paired t-test

Parameters	Groups	Baseline (Mean ± SD)	3 months (Mean ± SD)	##p value
Probing depth (mm)	Group 1	9.13±2.68	7.13±1.36	0.000*
	Group 2	9.15±2.44	6.03±1.14	0.000*
#p value		0.78	0.03*	
Clinical attachment level (mm)	Group 1	8.37±1.43	6.57±0.55	0.000*
	Group 2	8.47±1.11	4.73±0.67	0.000*
#p value		0.31	0.001*	
Plaque index	Group 1	3.11±0.42	1.43±0.02	0.000*
	Group 2	3.65±0.45	1.43±0.32	0.000*
#p value		0.67	0.18	

## Discussion

A multifaceted conflict between a bacterial biofilm and a vulnerable host results in periodontitis. The most popular non-surgical therapy strategy for reducing inflammation is SRP; however, this modality is unsuccessful in deep subgingival areas due to access restrictions [3]. Hence, auxiliary medicines are utilized in addition to SRP. Numerous studies have assessed the effects of various auxiliary agents, including non-chemical, chemical, antibiotic, and herbal agents [16,17]. In this study, the effects of aloe vera hydrogel application in patients with chronic periodontitis as a supplement to SRP were assessed.

In this study, aloe vera extract was made in the form of hydrogel. Hydrogels, which are made of three-dimensional viscoelastic networks, are hydrophilic polymers that swell and hold water many times their dry weight under physiological conditions [18]. The hydrogel has high water content, which is primarily responsible for its biocompatibility, and its extracellular matrix-like physicochemical and mechanical properties make it the perfect material for dressing wounds. Additionally, hydrogels are adaptable to the kind of surface they are applied to and are reasonably deformable. For a variety of biomedical applications, the latter qualities combined with the bio-/mucoadhesive characteristic of hydrogels can be used to keep them contained at the application site [19].

Both the case and control groups' periodontal indices were examined and compared in this study. The findings revealed that, when aloe vera was combined with SRP, there was a significant improvement in the clinical attachment level and probing depth. This may be because biologically active substances such as mannose 6-phosphate, carboxypeptidase, glutathione peroxidase, and superoxide dismutase are found in aloe vera gel. These substances have anti-inflammatory, antibacterial, and antioxidant qualities that support wound healing and immune system regulation [20].

Singh et al. [21] evaluated the efficacy of aloe extract in periodontal diseases and demonstrated that there was more reduction in bleeding scores and probing depth values when aloe was used along with mechanical debridement. Furthermore, a clinical trial reported that aloe vera, when used along with SRP as local drug delivery, reduced gingival inflammation and probing depth. Additionally, it has been reported that locally delivered aloe vera gel resulted in more gain in CAL in diabetic patients with periodontitis [22]. These results are in line with the current investigation.

Limitations

The primary constraint of the current investigation was its short follow-up and minimal sample size; consequently, it is recommended to carry out studies using different vehicles for delivering the drug among larger populations with long-term follow-ups to substantiate the findings. Additionally, this study failed to determine the optimal dosage, bioavailability, and the effect of frequency of application.

## Conclusions

According to this study, aloe vera hydrogel in combination with SRP in generalized chronic periodontitis patients, resulted in improving the periodontal parameters. Therefore, the study data suggest aloe vera as a potential adjunct to periodontal therapy. However, randomized controlled clinical trials are warranted to assess the efficacy of other modes of delivering aloe vera, optimal dosage, and its effect on microbiological and immunological parameters with long-term follow-ups to substantiate these findings.
